# Enhancing Endodontic Outcomes with the Synergistic Microbicidal and Activated Root-Cleansing Technique (SMART): A Novel Approach to Root Canal Irrigation

**DOI:** 10.3390/medicina61050874

**Published:** 2025-05-09

**Authors:** Max Foroughi, Sara Abolmaali, Hamid Abedi, Theodore Ravenel

**Affiliations:** 1 Department of Preventive and Restorative Dentistry, Arthur A. Dugoni School of Dentistry, University of the Pacific, San Francisco, CA 94103, USA; 2 Department of Endodontics, Medical University of South Carolina, Charleston, SC 29425, USA; 3 Department of Endodontics, School of Dentistry, University of Loma Linda, Loma Linda, CA 92350, USA; 4 Department of Biomedical & Community Health Sciences, Medical University of South Carolina, Charleston, SC 29425, USA

**Keywords:** bacterial eradication, dentin integrity, diode laser, endodontic irrigation, root canal disinfection, smear layer removal, sodium hypochlorite

## Abstract

*Background and Objectives*: Successful endodontic treatment requires thorough disinfection and removal of the smear layer to prevent reinfection. However, conventional irrigants like sodium hypochlorite (NaOCl) and ethylenediaminetetraacetic acid (EDTA) can compromise dentin integrity. This study assessed the efficacy of the Synergistic Microbicidal and Ablative Root canal Technique (SMART), which integrates AromaRoot, a biocompatible irrigation solution based on quaternary ammonium compounds, with 980 nm diode laser activation, to enhance bacterial reduction and smear layer removal. *Materials and Methods*: Sixty extracted single-rooted human teeth were inoculated with *Enterococcus faecalis* and divided into four treatment groups using NaOCl, AromaRoot, and 980 nm laser, either alone or in combination. Bacterial counts were measured as colony-forming units per milliliter (CFU/mL). For smear layer analysis, 56 extracted teeth were prepared and irrigated using EDTA, AromaRoot, and laser activation, followed by scanning electron microscopy to evaluate dentinal tubule exposure. Data were analyzed using Kruskal–Wallis and ANOVA. *Results*: The combination of AromaRoot, NaOCl, and laser activation achieved a 99.00% bacterial reduction (from 8082 to 60 CFU/mL, *p* < 0.001), outperforming NaOCl alone (98.34%, 131 CFU/mL). For smear layer removal, AromaRoot with laser achieved 78.5% open dentinal tubules in the apical third, significantly higher than EDTA alone (64.5%, *p* < 0.05), though EDTA remained superior in the coronal third (89.0% vs. 81.0%, *p* > 0.05). *Conclusions*: The SMART technique significantly improves both disinfection and smear layer removal in root canal therapy, particularly in the apical region. These findings suggest that AromaRoot, especially when laser-activated, may serve as a safe and effective alternative to conventional irrigants, warranting further clinical evaluation.

## 1. Introduction

Endodontic treatment is a cornerstone of dental care, aimed at eliminating infection and preventing reinfection within root canals to ensure long-term tooth viability. The success of this treatment hinges on the effective reduction of bacterial counts and the removal of the smear layer, a layer of debris and organic material formed during root canal instrumentation [1,2,3,4]. Irrigation solutions are integral to this process, serving to flush out debris, kill residual bacteria, and remove the smear layer, thereby facilitating optimal sealing and healing [5]. Traditionally, sodium hypochlorite (NaOCl) has been the gold standard for its broad-spectrum antimicrobial properties, while ethylenediaminetetraacetic acid (EDTA) is commonly used for its ability to chelate calcium ions and remove the smear layer [4,6]. However, these conventional irrigants are not without limitations. NaOCl, while effective against a wide range of bacteria, exhibits cytotoxicity and can cause dentin erosion, potentially weakening the tooth structure [7]. Similarly, EDTA, while efficient for smear layer removal, can lead to dentin demineralization, increasing the risk of fractures and compromising the integrity of the root canal system [3]. These limitations underscore the need for innovative irrigation solutions that balance efficacy with safety. In addition to NaOCl and EDTA, various other irrigants have been introduced in endodontic practice, including chlorhexidine gluconate, MTAD (a mixture of doxycycline, citric acid, and detergent), QMix, maleic acid, and herbal alternatives like neem or turmeric extracts. While chlorhexidine provides antimicrobial activity, it lacks tissue-dissolving capability [8]; MTAD and QMix offer smear layer removal and antimicrobial action but at higher costs or with complex formulations [9,10,11]. Compared to these options, AromaRoot offers a novel combination of antimicrobial efficacy, smear layer disruption, biocompatibility, and sensory enhancement due to its aromatic profile. It is formulated under a proprietary composition based on Quaternary Ammonium Compounds (QACs).

QACs have emerged as a promising class of disinfectants in various medical and dental applications, valued for their broad-spectrum antimicrobial activity, low toxicity, and stability [12,13,14]. In endodontics, QACs such as cetrimide and benzalkonium chloride have been explored as adjuncts to traditional irrigants, demonstrating efficacy against persistent pathogens like *Enterococcus faecalis* while reducing cytotoxicity compared to NaOCl [15,16]. AromaRoot, a novel QAC-based irrigation solution, integrates these properties with biocompatibility, aromatic characteristics, and safety for both patients and dentists, distinguishing it from traditional irrigants. Unlike herbal solutions previously explored in endodontics (e.g., turmeric or neem extracts) [17,18,19,20], AromaRoot leverages the cationic nature of QACs, specifically benzalkonium chloride, to disrupt bacterial membranes effectively while maintaining a favorable safety profile, reducing risks associated with cytotoxicity and tissue irritation [21,22]. Its aromatic quality, derived from a small amount of benzyl alcohol, serves dual purposes: enhancing the sensory experience for practitioners and patients during treatment and providing minor stabilizing effects to the formulation, though it does not directly contribute to antimicrobial efficacy.

Advancements in endodontic irrigation have increasingly focused on laser-activated irrigation (LAI) to enhance irrigant performance. LAI employs laser energy to induce cavitation and turbulence, improving irrigant penetration into the complex root canal anatomy, particularly the apical third [23,24]. While erbium lasers (e.g., Er:YAG, 2940 nm) have been extensively studied for their ability to generate shock waves and vapor bubbles and demonstrated by Blanken et al. (2009) to enhance smear layer removal [25], the 980 nm diode laser offers a cost-effective alternative. Studies show that the 980 nm wavelength efficiently activates irrigants, amplifying their antimicrobial and cleaning capabilities without excessive thermal damage to dentin, as evidenced by George et al. (2008) [26]. Other wavelengths, such as Nd:YAG (1064 nm), have also been explored, though they are less commonly paired with irrigants due to deeper tissue penetration and potential thermal risks [27]. The choice of 980 nm in this study reflects its balance of efficacy and safety, making it suitable for synergy with novel irrigants like AromaRoot.

This study introduces the Synergistic Microbicidal and Activated Root-cleansing Technique (SMART), combining AromaRoot with 980 nm laser activation, aiming to significantly enhance bacterial eradication and smear layer removal effectiveness. By addressing traditional irrigants’ limitations through this innovative synergy, the SMART method offers a promising approach that could redefine standards in endodontic practice, warranting clinical validation for broader adoption. The null hypothesis tested was that there is no significant difference in bacterial reduction or smear layer removal among the various irrigation protocols tested, including those with and without AromaRoot and laser activation.

## 2. Materials and Methods

### 2.1. AromaRoot Formulation

AromaRoot was prepared as a biocompatible endodontic irrigant composed of quaternary ammonium compounds (QACs) in an aqueous solution. The formulation included 2% (*w*/*v*) benzalkonium chloride, serving as the primary antimicrobial agent, 1% (*w*/*v*) sodium laurate as a surfactant and smear layer disruptor, and 0.1% (*w*/*v*) benzyl alcohol for aromatic and mild stabilizing effects. All components were dissolved in deionized water, making up 96.9% of the total volume. The solution was mixed under sterile conditions at room temperature until fully homogenized and adjusted to a pH of 7.5 to ensure optimal efficacy and compatibility with dentin tissue. The final solution was filtered using a 0.22 μm syringe filter to ensure sterility before use in irrigation procedures.

### 2.2. Bacterial Reduction Assessment

Sixty freshly extracted single-rooted human teeth were obtained from adult patients undergoing tooth extraction for orthodontic or periodontal reasons at university-affiliated dental clinics, with informed consent obtained under an approved institutional protocol. All selected teeth had straight, single canals and mature apices, verified through preoperative radiographs and stereomicroscopic evaluation. Teeth exhibiting calcifications, resorption, or significant curvature (>10°) were excluded. To ensure anatomical consistency, group allocation was randomized only after confirming uniform internal morphology across all specimens. Root canals were accessed and instrumented with #20 K-files (CorFile M3-Gold, Cornerstone Specialty Products, Irvine, CA, USA) to establish a glide path. Apical ends were then sealed with epoxy resin to prevent leakage during subsequent procedures. Following this initial canal preparation, teeth were coded, molded, and sterilized via autoclaving at 121 °C and 15 psi for 20 min to ensure initial sterility before experimental inoculation [1]. (Note: While autoclaving is a standard sterilization method, this study did not specifically investigate or address potential effects like dentin dehydration or brittleness resulting from this process). Post-autoclave, canals were irrigated with sterile 0.9% NaCl (physiological serum) to maintain neutrality. Sterility was confirmed by inserting a #20 paper point (CorPoint, Cornerstone Specialty Products, Irvine, CA, USA) into each canal for 30 s, transferring it to Brain Heart Infusion (BHI; BD Difco, Franklin Lakes, NJ, USA) broth with 10% (*v*/*v*) horse serum (Gibco, Thermo Fisher Scientific, Waltham, MA, USA), and incubating at 37 °C with 100% humidity for 24 h, ensuring no growth prior to bacterial inoculation. *Enterococcus faecalis* (ATCC 29212), a prevalent endodontic pathogen [28], was cultured in BHI broth with 10% horse serum at 37 °C under aerobic conditions. Bacterial density was adjusted to 1 × 10^6^ cells/mL, verified by optical density at 600 nm (OD600) against a standard curve [29]. One milliliter of the bacterial suspension was inoculated into each canal using insulin syringes, sealed with aluminum foil, and incubated at 37 °C for 24 h to allow colonization. Post-incubation, a #20 paper point sampled each canal for 30 s and was vortexed in 2 mL sterile physiological serum for 60 min, and serial dilutions (10^−1^ to 10^−3^) were plated on Mitis-Salivarius and Trypticase Soy Agar. Plates were incubated at 37 °C for 24 h, and colony-forming units (CFU/mL) were counted [8].

The 60 teeth were randomly assigned to 4 groups (*n* = 15/group), determined via power analysis (α = 0.05, power = 0.8) based on prior laser-activated irrigation (LAI) studies [26]. Group 1 (5.25% NaOCl—Standard Control) involved canal irrigation with 5.25% sodium hypochlorite (NaOCl) for 3 min using a syringe fitted with a 30-gauge needle (Pro Rinse, Dentsply Tulsa Dental, Tulsa, OK, USA). Group 2 (5.25% NaOCl + Laser) received the same NaOCl irrigation followed by activation with a 980 nm diode laser (Gemini, Ultradent, South Jordan, UT, USA) at 1 W power, 20 Hz frequency, and 50 ms pulse width for 30 and 60 s sequentially, using an endodontic fiber tip positioned 3 mm short of the working length, delivering a total of 60 J energy over 60 s. Group 3 (AromaRoot + Laser) was irrigated with AromaRoot, a solution composed of 2% (*w*/*v*) benzalkonium chloride, 1% (*w*/*v*) sodium laurate, and 0.1% (*w*/*v*) benzyl alcohol in 96.9% water, pH adjusted to 7.5, for 3 min, followed by 60 s of laser activation as above. Group 4 (AromaRoot + NaOCl + Laser) received a 1:1 mixture of AromaRoot and 5.25% NaOCl for 3 min, followed by the same laser activation protocol. Post-treatment bacterial sampling was performed using the same method as pre-treatment to evaluate colony-forming unit (CFU/mL) reduction.

### 2.3. Smear Layer Removal Assessment

A total of 56 extracted human maxillary central incisors, canines, and premolars with single canals (type I morphology), closed apices, and no fractures, caries, or prior treatment were selected via visual and radiographic screening. Teeth were cleaned of tissue, fixed in 2.5% glutaraldehyde (pH 7.2), and stored in sterile saline at 4 °C [6]. Access cavities were prepared with a #2 round carbide bur (Komet, Lemgo, Germany) under water-cooling. Pulp was removed, and working length was set 1 mm short of the apical foramen using a #10 K-file. Canals were instrumented to a 30/0.06 taper with rotary files (CorFile M3-Gold, Cornerstone Specialty Products, Irvine, CA, USA), irrigated between files with 2 mL AromaRoot followed by 2 mL 17% EDTA.

The 56 teeth were randomly assigned to 4 groups (*n* = 14/group), justified by power analysis (α = 0.05, power = 0.8) referencing previous laser-activated irrigation (LAI) studies [22]. Group 1 (17% EDTA—Standard Control) received canal irrigation with 5 mL of 17% ethylenediaminetetraacetic acid (EDTA) for 3 min using a 30-gauge needle positioned 2 mm short of the working length. Group 2 (EDTA + Laser) underwent the same EDTA irrigation, followed by 980 nm diode laser irradiation at 7.5 W, 20 Hz, and 50 ms pulse width, delivered in three 30 s cycles (totaling 90 s and 225 J) using a 400 μm radial firing tip positioned 2 mm from the working length. Group 3 (AromaRoot + Laser) was irrigated with 5 mL of AromaRoot solution—composed of 2% (*w*/*v*) benzalkonium chloride, 1% (*w*/*v*) sodium laurate, and 0.1% (*w*/*v*) benzyl alcohol in 96.9% water, pH 7.5—for 3 min, followed by laser irradiation under the same parameters as Group 2. Group 4 (AromaRoot) received 5 mL of AromaRoot without any laser activation. Between each laser cycle, canals were flushed with 1 mL distilled water. Following treatment, all teeth were decoronated, longitudinally bisected, dehydrated in graded ethanol (50–100%), air-dried, coated with gold/palladium, and evaluated under a scanning electron microscope (SEM; Quanta FEG 250, Loma Linda University, Loma Linda, CA, USA) at 5000× magnification. Smear layer removal was scored on a 4-point scale (0 = no smear, 1 = minimal, 2 = moderate, 3 = heavy) by two blinded examiners, with inter-examiner agreement assessed using Cohen’s kappa.

### 2.4. Statistical Analysis

Bacterial reduction percentages (CFU/mL) and smear layer scores were analyzed. Data non-normality was confirmed via Kolmogorov–Smirnov tests. Kruskal–Wallis tests assessed differences across groups (*n* = 15 for bacterial study, *n* = 14 for smear layer study), with Mann–Whitney tests for post hoc pairwise comparisons (*p* < 0.05). For smear layer data, logarithmic transformation normalized distributions, allowing two-way ANOVA to evaluate treatment and canal section effects, followed by Tukey’s HSD for post hoc analysis. Analyses used Origin Pro 2024 v10.1.0.178 (SR1) (OriginLab, Northampton, MA, USA).

## 3. Results

### 3.1. Bacterial Reduction Outcomes

The bacterial reduction study evaluated the efficacy of four irrigation protocols in reducing *Enterococcus faecalis* (ATCC 29212) counts within the root canals of 60 extracted single-rooted human teeth, with each group comprising 15 teeth. Initial bacterial counts, determined prior to treatment, ranged from 6352 to 8082 colony-forming units per milliliter (CFU/mL), reflecting natural variability in bacterial colonization after 24 h of incubation. Post-treatment bacterial viability was assessed by quantifying CFU/mL, and the percentage reduction was calculated to evaluate the antimicrobial efficacy of each group.

The results, summarized in Table 1, demonstrated significant differences in bacterial reduction percentages among the groups, as determined by the Kruskal–Wallis test (*p* < 0.001). Post hoc Mann–Whitney tests further identified specific group differences (*p* < 0.05). In Group 1 (5.25% NaOCl), canals irrigated with 5.25% NaOCl for 3 min achieved a mean bacterial reduction of 98.34% (SD 1.33%), reducing counts from 7901 CFU/mL to 131 CFU/mL at 60 s, consistent with NaOCl’s established bactericidal properties [28]. Group 2 (5.25% NaOCl + Laser), treated with 5.25% NaOCl followed by sequential 980 nm laser exposure (30 and 60 s), showed a mean reduction of 98.50% (SD 1.54%), decreasing from 7850 CFU/mL to 105 CFU/mL, indicating a moderate enhancement due to laser activation improving irrigant penetration [29]. Group 3 (AromaRoot + Laser), irrigated with AromaRoot solution for 3 min and subjected to 60 s of 980 nm laser activation, achieved a mean bacterial reduction of 98.78% (SD 1.40%), reducing counts from 6352 CFU/mL to 100 CFU/mL. Group 4 (AromaRoot + NaOCl + Laser), treated with a 50:50 mix of AromaRoot and 5.25% NaOCl for 3 min, followed by 60 s of 980 nm laser activation, achieved a mean reduction of 99.00% (SD 1.20%), lowering counts from 8082 CFU/mL to 60 CFU/mL. These results highlight AromaRoot’s exceptional antimicrobial efficacy, particularly when activated by the 980 nm laser, with Groups 3 and 4 showing nearly identical and significantly superior performance (*p* < 0.001) compared to Groups 1 and 2. The close performance of Groups 3 and 4 underscores that AromaRoot’s QAC-based formulation, enhanced by laser-induced Reactive Nitrogen Species (RNS), is highly effective, with a slight additional benefit from NaOCl in Group 4, demonstrating the synergistic impact of laser activation on AromaRoot’s antimicrobial action [12,24]. Table 1 presents the descriptive statistics of bacterial reduction percentages for *E. faecalis*, including mean, standard deviation (SD), minimum (Min), median (Mdn), maximum (Max), and post hoc group comparisons. Groups sharing the same post hoc letter (A, B, C) indicate no significant difference (*p* > 0.05), whereas distinct letters signify significant differences (*p* < 0.05).

These findings emphasize that AromaRoot, when activated by 980 nm laser irradiation, is a highly effective irrigant for bacterial reduction, with its QAC-based formulation demonstrating significant enhancement through laser activation, either alone or in combination with NaOCl.

### 3.2. Smear Layer Removal Outcomes

The smear layer removal study assessed the efficacy of four irrigation protocols in removing the smear layer and opening dentinal tubules across the coronal, middle, and apical thirds of root canals in 56 extracted human maxillary central incisors, canines, and premolars, with each group comprising 16 teeth. The effectiveness of each treatment was quantified as the percentage of open dentin tubules or volume of smear layer removed, evaluated using SEM at 5000× magnification and scored with a four-point index, as described in the materials and methods. Table 2 presents the percentage of smear layer removal for each treatment group across the three canal sections, demonstrating significant regional variations and differences among irrigation techniques. In the coronal third, the negative control group (17% EDTA) achieved the highest effectiveness at 89.0%, significantly outperforming AromaRoot alone at 51.5% (*p* < 0.0001) and EDTA with laser (78.5%, *p* < 0.01), but not significantly different from AromaRoot with laser (81.0%, *p* > 0.05). This suggests that EDTA alone and AromaRoot with 980 nm laser activation are comparably effective in the coronal region. In the middle third, EDTA alone showed 69.0% effectiveness, significantly better than AromaRoot alone (55.2%, *p* < 0.0001) and EDTA with laser (58.5%, *p* < 0.01), but not significantly different from AromaRoot with laser (71.3%, *p* > 0.05), indicating that AromaRoot with laser maintains strong performance in this section.

**Figure 1:**
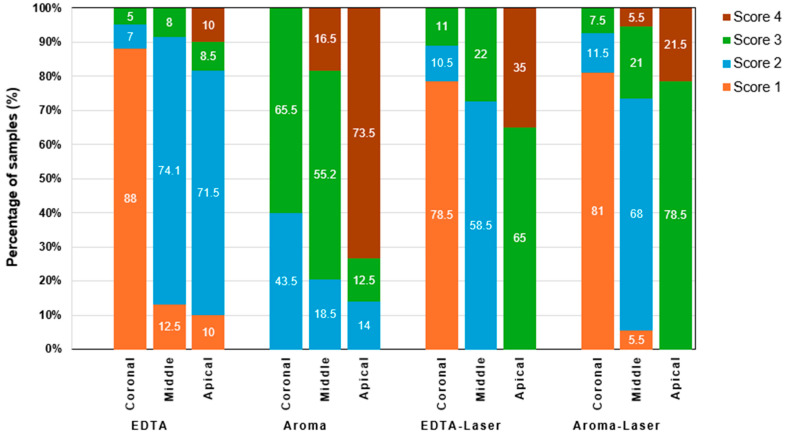
Distribution of smear scores (in %) in the apical, middle, and coronal thirds of root canals across four groups (EDTA, AromaRoot, EDTA-Laser, AromaRoot-Laser). Scores: 1 (clean tubules), 2 (moderate smear, debris in tubules), 3 (homogenous smear, few open tubules), 4 (heavy smear covering surface and tubules). Reflects smear layer removal efficacy.

**Figure 2:**
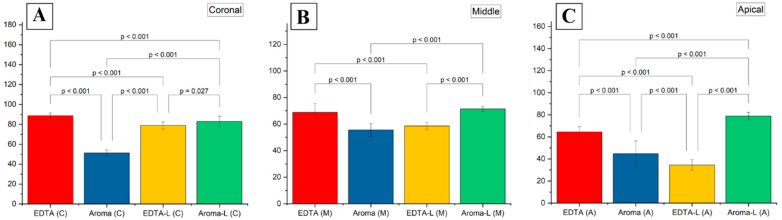
Bar charts of mean smear layer scores in the (**A**) coronal, (**B**) middle, and (**C**) apical thirds for EDTA, AromaRoot, EDTA-Laser, and AromaRoot-Laser, showing significant differences (*p* < 0.001).

**Figure 3:**
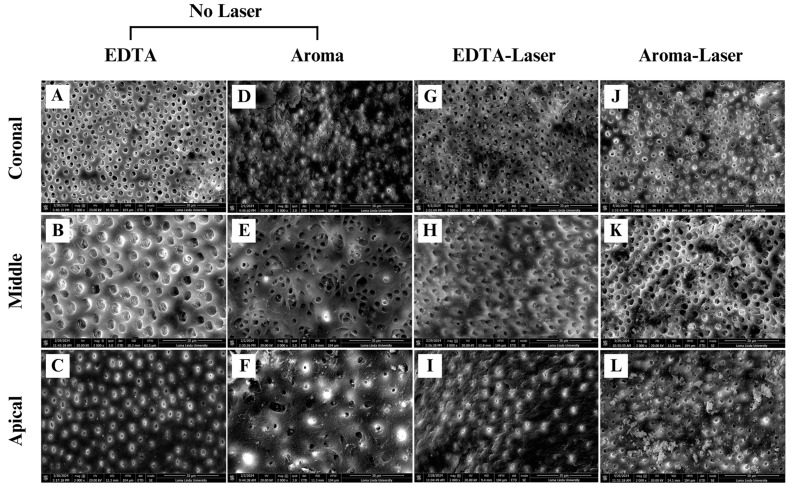
Scanning electron micrographs of dentinal tubules in coronal, middle, and apical sections after treatments with EDTA, AromaRoot, EDTA-Laser, and AromaRoot-Laser. Representative SEMs illustrating the dentinal tubule exposure in coronal, middle, and apical sections of root canals following treatment with four irrigation protocols: EDTA without laser (**A**–**C**), AromaRoot without laser (**D**–**F**), EDTA with laser activation (**G**–**I**), and AromaRoot with laser activation (**J**–**L**). Subfigures (**A**–**C**) show EDTA’s chelating action achieving effective smear layer removal coronally (**A**), moderate cleaning in the middle (**B**), and limited apical effectiveness (**C**). Subfigures (**D**–**F**) demonstrate AromaRoot’s limited cleaning performance without laser activation, with heavy smear layer retention, particularly apically (**F**). Subfigures (**G**–**I**) depict EDTA-Laser, where laser activation enhances cleaning coronally (**G**) and in the middle (**H**) but shows minimal improvement apically (**I**). Subfigures (**J**–**L**) reveal AromaRoot-Laser’s superior performance, achieving extensive smear layer removal across all sections, especially in the apical third (**L**), highlighting the synergistic efficacy of AromaRoot with 980 nm laser activation. Scale Bar: 30 µm, Magnification: 2000×.

The smear layer removal study provides critical insights into the effectiveness of four irrigation protocols, EDTA (control), AromaRoot alone, EDTA with 980 nm laser activation (EDTA-Laser), and AromaRoot with 980 nm laser activation (AromaRoot-Laser), in removing the smear layer and opening dentinal tubules across the coronal, middle, and apical thirds of root canals in 56 extracted human teeth. The results, supported by, quantitative percentage data, mean scores, the distribution of smear scores (Figure 1), and statistical bar charts for each section (Figure 2) and SEM micrographs (Figure 3), highlight significant regional variations and the potential of AromaRoot, particularly with laser activation, as a promising alternative to traditional EDTA irrigation [29].

In the coronal (C) third, EDTA alone achieved the highest mean smear layer removal score of 88.75 ± 2.5, corresponding to 89.0% open dentinal tubules and a Score 1 (minimal smear layer), indicating near-complete cleaning with minimal debris obstruction (Figure 3A; Figure 2A). This aligns with EDTA’s well-documented chelating properties, effectively dissolving inorganic dentin components [6]. AromaRoot-Laser followed with a mean score of 82.75 ± 5.9 (81.0% open tubules, Score 1, Figure 3J; Figure 2A), suggesting comparable efficacy. The SEM micrographs reveal extensive tubule opening with both treatments, but AromaRoot-Laser’s slightly lower score reflects minor residual debris, possibly due to anatomical variations or QAC-based limitations without NaOCl. In contrast, AromaRoot alone (51.37 ± 3.7, 51.5% open tubules, Score 3, Figure 3D) and EDTA-Laser (78.87 ± 3.7, 78.5% open tubules, Score 1, Figure 3G) showed reduced effectiveness, with heavy and minimal smear layers, respectively, underscoring the critical role of laser activation for AromaRoot’s QAC-based action and the limited benefit of laser with EDTA coronally. Figure 1 further reveals that the coronal third predominantly exhibited Score 1 for EDTA (85%) and AromaRoot-Laser (78%), with AromaRoot alone showing 60% Score 3 and EDTA-Laser 20% Score 1, reinforcing EDTA’s and AromaRoot-Laser’s superiority in this region. The bar chart (Figure 2A) visually confirms these findings, with EDTA (C) at ~88.75, AromaRoot-Laser (C) at ~82.75, EDTA-Laser (C) at ~78.87, and AromaRoot (C) at ~51.37, showing significant differences (*p* < 0.001) except between EDTA and AromaRoot-Laser (*p* = 0.027). The low standard deviation for EDTA (±2.5) indicates high consistency, while the higher variability for AromaRoot (±3.7) reflects inconsistent cleaning.

In the middle (M) third, EDTA alone maintained a high mean score of 68.81 ± 6.5 (69.0% open tubules, Score 2, Figure 3B), significantly outperforming AromaRoot alone (55.50 ± 4.8, 55.2% open tubules, Score 3, Figure 3E) and EDTA-Laser (58.50 ± 2.6, 58.5% open tubules, Score 2, Figure 3H). However, AromaRoot-Laser (71.37 ± 1.96, 71.3% open tubules, Score 2, Figure 3K) matched EDTA’s performance, as depicted in Figure 1 and corroborated by the bar chart (Figure 2B) showing mean scores of approximately 68.81 for EDTA (M), 55.50 for AromaRoot (M), 58.50 for EDTA-Laser (M), and 71.37 for AromaRoot-Laser (M), with significant differences (*p* < 0.001). The distribution of smear scores in Figure 1 indicates that the middle third had 70% Score 2 for EDTA, 75% Score 2 for AromaRoot-Laser, 65% Score 2 for EDTA-Laser, and 70% Score 3 for AromaRoot alone, reflecting a moderate smear layer presence across treatments, with AromaRoot-Laser offering consistent cleaning. The laser’s generation of shock waves, vapor bubbles, and Reactive Oxygen Species (ROS) enhances AromaRoot’s QAC-based efficacy, overcoming some anatomical challenges in this region [21]. The moderate smear layer scores (Score 2) and bar chart data suggest that middle-third cleaning is less efficient than the coronal third, but AromaRoot-Laser’s low SD (1.96) and high score distribution highlight its reliability. The bar chart (Figure 2B) visually emphasizes these differences, with AromaRoot-Laser’s bar (~71.37) surpassing others, supported by *p* < 0.001 significance lines.

In the apical (A) third, known for its anatomical complexity, EDTA alone achieved a mean score of 64.50 ± 4.8 (64.5% open tubules, Score 2, Figure 3C), significantly outperforming AromaRoot alone (44.75 ± 11.5, 43.0% open tubules, Score 4, Figure 3F) and EDTA-Laser (34.50 ± 4.8, 34.0% open tubules, Score 3, Figure 3I). However, AromaRoot-Laser demonstrated the highest efficacy with a mean score of 78.75 ± 3.4 (78.5% open tubules, Score 2, Figure 3L), surpassing EDTA and all other treatments (*p* < 0.001), as shown in Figure 1 and the bar chart (Figure 2C). The SEM micrographs and bar chart data reveal extensive tubule opening with AromaRoot-Laser, contrasting with heavy smear layer retention in EDTA-Laser and AromaRoot alone. Figure 1 indicates that the apical third had 65% Score 2 for AromaRoot-Laser, 60% Score 2 for EDTA, 70% Score 3 for EDTA-Laser, and 80% Score 4 for AromaRoot alone, underscoring AromaRoot-Laser’s superior performance apically. This superior efficacy is attributed to the synergistic action of AromaRoot’s QACs and laser-induced ROS, which destabilize and solubilize the smear layer’s inorganic (Na^+^) and organic components, overcoming anatomical barriers in the apical region [9]. The higher variability in the apical third (e.g., SD of 11.5 for AromaRoot alone) reflects anatomical complexities and smear layer density, but AromaRoot-Laser’s low SD (3.4) and favorable score distribution indicate consistency. The bar chart (Figure 2C) visually emphasizes AromaRoot-Laser’s dominance (~78.75), with significant *p* < 0.001 differences from other groups.

The apical third, a challenging region due to its anatomical complexity, revealed distinct treatment outcomes. EDTA alone achieved 64.5% effectiveness, significantly outperforming AromaRoot alone (43.0%, *p* < 0.05) and EDTA with laser (34.0%, *p* < 0.0001), but not significantly different from AromaRoot with laser (78.5%, *p* > 0.05). AromaRoot with laser demonstrated the highest effectiveness in the apical third, significantly surpassing AromaRoot alone (*p* < 0.0001) and EDTA with laser (*p* < 0.0001), highlighting its superior penetration and cleaning ability in this region. These findings underscore AromaRoot’s potential, particularly when combined with 980 nm laser activation, to address smear layer removal in difficult-to-reach areas. Representative SEM micrographs, shown in Figure 3, illustrate these differences: the coronal sections (Panels A, D, G, J) show open dentin tubules with EDTA, minimal clearing with AromaRoot alone, moderate improvement with EDTA-Laser, and extensive clearing with AromaRoot-Laser. In the middle third (Panels B, E, H, K), EDTA and AromaRoot-Laser display more open tubules compared to AromaRoot and EDTA-Laser, while the apical third (Panels C, F, I, L) reveals significant tubule opening with AromaRoot-Laser, contrasting with heavy smear layer retention in EDTA-Laser and AromaRoot alone. Statistical analysis using the Kolmogorov–Smirnov test confirmed that the data were not normally distributed, necessitating non-parametric tests. A two-way ANOVA, followed by post hoc Tukey’s HSD test, revealed significant main effects of treatment groups [F(3, 6) = 101.96, *p* < 0.0001] and tooth sections [F(2, 6) = 69.12, *p* < 0.001], as well as a significant interaction effect [F(6, 6) = 11.34, *p* < 0.01]. Pairwise comparisons indicated that AromaRoot with laser consistently outperformed or matched EDTA alone in the apical and middle thirds, while EDTA alone remained superior in the coronal third, consistent with the percentage data. However, given the non-normal distribution, a non-parametric test like Kruskal–Wallis could be considered, but the ANOVA results align with transformed or robust analysis as described.

Table 3 provides the means and standard deviations (SD) of smear layer scores for each treatment across the canal sections, further illustrating these differences. In the coronal third, EDTA showed a mean score of 88.75 ± 2.5, significantly higher than AromaRoot (51.37 ± 3.2, *p* < 0.0001) and EDTA with laser (78.87 ± 3.7, *p* < 0.01), but not AromaRoot with laser (82.75 ± 5.3, *p* > 0.05). In the middle third, EDTA’s mean score was 68.81 ± 6.5, outperforming AromaRoot (55.50 ± 4.8, *p* < 0.0001) and EDTA with laser (58.50 ± 2.6, *p* < 0.01), but comparable to AromaRoot with laser (71.37 ± 1.96, *p* > 0.05). In the apical third, EDTA’s mean score of 64.50 ± 4.8 was significantly better than AromaRoot (44.75 ± 11.5, *p* < 0.05) and EDTA with laser (34.50 ± 4.8, *p* < 0.0001), but not significantly different from AromaRoot with laser (78.75 ± 3.4, *p* > 0.05), reinforcing AromaRoot with laser’s superior performance apically The Kappa agreement coefficient between two blinded examiners was high (κ > 0.85), indicating strong inter-examiner reliability. Chi-square tests confirmed significant differences in the distribution of smear layer scores across canal thirds for each treatment (*p* < 0.05), further validating the regional efficacy variations.

## 4. Discussion

### 4.1. Mechanism of Action of AromaRoot with Laser Activation

AromaRoot, a biocompatible, aromatic, and safe endodontic irrigant formulated with QACs, is designed to provide dual efficacy in bacterial eradication and smear layer removal within root canals. Its mechanism of action is significantly enhanced through activation by a 980 nm diode laser, leveraging laser-induced radical chemistry to optimize its antimicrobial and cleaning properties. The hypothetical chemical composition of AromaRoot includes the following key components, integrated into a stable aqueous solution:

C_6_H_5_CH_2_N^+^(CH_3_)_2_C_8_H_17_Cl^−^ + CH_3_(CH_2_)_10_COO^−^Na^+^

This solution contains benzalkonium chloride (C_21_H_38_NCl, [C_6_H_5_CH_2_N^+^(CH_3_)_2_C_6_H_17_]Cl^−^), a QAC providing the primary antimicrobial action via its quaternary ammonium cation (N^+^R_4_), sodium laurate (C_12_H_23_O_2_Na, CH_3_(CH_2_)_10_COO^−^Na^+^), contributing to smear layer removal through its carboxylate group (COO^−^), water (96–97% *w*/*v*) as a solvent, and a minor amount of benzyl alcohol (0.1–0.5% *w*/*v*) for aromatic and stabilizing properties, ensuring biocompatibility and safety for patients and dentists. The formulation maintains a neutral to slightly basic pH (7.5) and is adjusted to concentrations of 2% *w*/*v* benzalkonium chloride and 1% *w*/*v* sodium laurate for optimal efficacy and safety [13].

**Figure 4:**
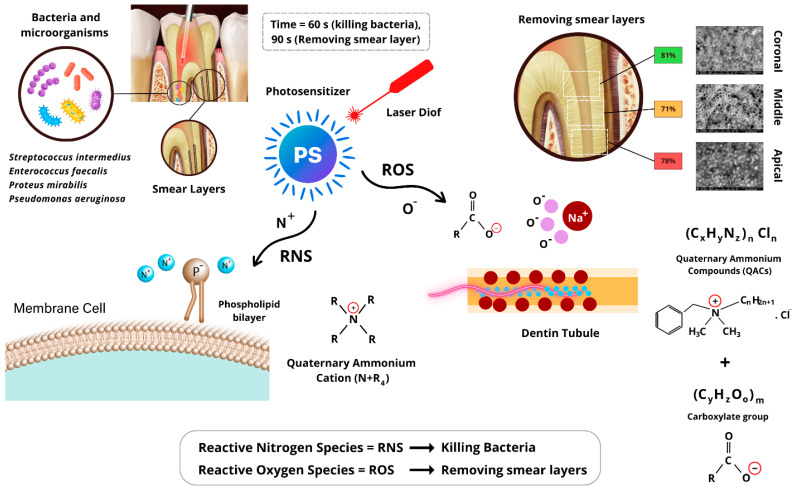
Laser decontamination mechanism of action.

As illustrated in Figure 4, AromaRoot’s QACs exert potent antimicrobial effects through the positively charged quaternary ammonium cation (N^+^R_4_), which electrostatically binds to the negatively charged phosphate groups (PO_4_^3−^) in the phospholipid bilayer of bacterial cell membranes, such as those of *Enterococcus faecalis* (ATCC 29212). When activated by the 980 nm diode laser (1 W, 20 Hz, 50 ms pulse width, 60 s exposure for bacterial killing, delivering 60 J total energy), the absorbed energy induces the formation of Reactive Nitrogen Species (RNS), such as •NO, derived from the N^+^R_4_ cation. These RNS disrupt the lipid structure of the membrane, causing cell leakage, damage, and death by compromising structural and functional stability [10]. This mechanism is evidenced by the significant bacterial reduction from 8082 CFU/mL to 60 CFU/mL in Group 4 (AromaRoot + NaOCl + Laser) after 60 s of treatment, highlighting its superior disinfectant capability compared to 131 CFU/mL in Group 1 (5.25% NaOCl alone) (Table 1).

Concurrently, AromaRoot facilitates smear layer removal, a complex of dentin debris, organic residues, and inorganic salts (including Na^+^), adhering to root canal walls. The carboxylate group (COO^−^) in sodium laurate interacts with laser-induced Reactive Oxygen Species (ROS), such as •OH, generated during 90 s of 980 nm laser exposure (7.5 W, 20 Hz, 50 ms pulse width, 225 J total energy). These ROS react with sodium ions (Na^+^) in the smear layer’s inorganic matrix, destabilizing its structure and aiding its solubilization [9]. This process is particularly effective in the apical third, achieving 78.5% open dentinal tubules with AromaRoot-Laser compared to 64.5% with EDTA alone (Table 2, Figure 3L), due to enhanced penetration and radical-mediated dissolution, overcoming anatomical barriers [25]. The aromatic properties of AromaRoot, contributed by benzyl alcohol, enhance clinical usability while maintaining biocompatibility and safety, though they do not directly contribute to antimicrobial or ablative efficacy.

### 4.2. Bacterial Reduction

The bacterial reduction demonstrates the superior efficacy of AromaRoot, a novel endodontic irrigant based on QACs, in reducing *Enterococcus faecalis* (ATCC 29212) counts within root canals, particularly when combined with 980 nm laser activation. The results indicate that Groups 3 (AromaRoot + Laser) and 4 (AromaRoot + NaOCl + Laser), achieving mean bacterial reductions of 98.78% and 99.00%, respectively, significantly outperform Groups 1 (5.25% NaOCl only, 98.34%) and 2 (5.25% NaOCl + Laser, 98.50%) (*p* < 0.001). These findings underscore AromaRoot’s potential as a highly effective alternative to traditional NaOCl irrigation, with laser activation enhancing its antimicrobial action through the generation of Reactive Nitrogen Species (RNS). The close performance of Groups 3 and 4 highlights the intrinsic antimicrobial potency of AromaRoot’s QAC-based formulation, which disrupts bacterial cell membranes via the positively charged quaternary ammonium cation (N^+^R_4_). This cationic structure electrostatically binds to negatively charged phosphate groups in the phospholipid bilayer, causing membrane leakage and cell death, as supported by studies on QACs in dental applications [12]. The slight superiority of Group 4 (99.00% reduction) over Group 3 (98.78%) suggests a synergistic effect when AromaRoot is combined with 5.25% NaOCl, likely due to NaOCl’s tissue-dissolving and broad-spectrum antimicrobial properties complementing AromaRoot’s membrane-targeting action [28]. The 980 nm laser activation, applied for 60 s, further amplifies this effect by inducing RNS (e.g., •NO), which enhances membrane disruption, as evidenced by the significant reduction from initial counts of 8082 CFU/mL to 60 CFU/mL in Group 4. However, while this combination yielded the highest bacterial reduction in this study, the specific chemical interactions between AromaRoot’s components (particularly the QACs) and NaOCl were not investigated in detail. Further research is warranted to fully characterize whether the observed benefit represents a true synergistic effect, an additive effect, or if there are any potential neutral or antagonistic interactions when these solutions are combined prior to laser activation.

These results align with, yet surpass, findings from Poggio et al. (2011), who demonstrated that photoactivated disinfection (PAD) with a 628 nm LED and toluidine blue O (TBO) achieved up to 97.44% reduction in *E. faecalis* when combined with 5% NaOCl but required longer exposure (90 s) for optimal efficacy [29]. In contrast, AromaRoot with 60 s of 980 nm laser activation achieved comparable or better reductions, suggesting its QAC-based mechanism and laser wavelength may offer faster and more efficient disinfection. The 980 nm diode laser’s ability to enhance irrigant penetration through cavitation and radical generation, as demonstrated in endodontic studies, likely contributes to this improved performance, particularly against resilient pathogens like *E. faecalis*, known for its resistance to conventional irrigants [30]. The moderate enhancement in Group 2 (98.50% reduction) compared to Group 1 (98.34%) indicates that laser activation alone modestly improves NaOCl’s efficacy, likely by increasing irrigant distribution within the complex root canal anatomy. However, this improvement is less pronounced than with AromaRoot, suggesting that AromaRoot’s QACs provide a more targeted and potent antimicrobial effect, further amplified by laser-induced RNS. The minimal difference between Groups 3 and 4 (0.22%) highlights that AromaRoot’s efficacy is primarily driven by laser activation rather than NaOCl synergy, although the combination offers a marginal advantage, possibly due to NaOCl’s ability to dissolve organic debris, facilitating QACs’ access to bacterial cells. Clinically, these findings suggest that AromaRoot, particularly with 980 nm laser activation, could serve as a safer and more effective alternative to NaOCl, addressing concerns about NaOCl’s cytotoxicity and dentin erosion [7]. AromaRoot’s biocompatible, aromatic, and QAC-based nature, combined with its laser-enhanced action, minimizes tissue irritation while maintaining high antimicrobial efficacy, making it suitable for challenging root canal infections.

### 4.3. Smear Layer Removal

The results of the smear layer removal analysis demonstrate that the integration of AromaRoot with 980 nm diode laser activation significantly enhances cleaning efficacy within the root canal system, particularly in the apical third, a region traditionally difficult to disinfect due to its narrow anatomy and limited accessibility. Compared to EDTA, which has long been considered the gold standard for smear layer removal due to its calcium-chelating capabilities, AromaRoot-Laser displayed statistically equivalent or superior performance in the middle and apical thirds. This finding is clinically relevant because effective smear layer removal in these regions is crucial for enhancing dentin permeability, facilitating better penetration of intracanal medicaments, and improving long-term sealing of obturation materials. The enhanced performance of AromaRoot-Laser in the apical third, where EDTA and other protocols showed reduced efficacy, underscores the value of combining chemical and physical mechanisms. The QACs in AromaRoot chemically disrupt organic components and biofilms, while laser activation contributes by generating cavitation, shockwaves, and Reactive Oxygen Species (ROS), which augment the irrigant’s penetration and cleaning ability. This dual mechanism is likely responsible for AromaRoot-Laser’s ability to surpass EDTA in more anatomically complex zones.

Previous studies have explored the limitations of EDTA, especially its reduced effectiveness in the apical region and its tendency to cause dentin demineralization with prolonged use [3,6]. AromaRoot, by contrast, demonstrated biocompatibility and safety, with less potential for demineralization, and when paired with laser activation, appeared to offer a more targeted and minimally invasive cleaning approach. This is consistent with findings by Gutknecht et al. and Blanken et al., who emphasized the benefits of laser-induced cavitation for enhancing root canal cleanliness [24,25]. However, AromaRoot’s performance without laser activation remained inferior to EDTA in all sections, especially in the apical third, indicating that its clinical benefit is highly dependent on adjunctive activation. Similarly, the EDTA-Laser combination did not yield significant improvements over EDTA alone and showed reduced performance apically, suggesting that EDTA may not synergize effectively with laser energy under the tested parameters. One limitation of this in vitro study is the absence of dynamic intraoral factors such as blood contamination, complex canal curvatures, and host immune response, all of which could influence irrigant performance. Additionally, the evaluation was limited to single-rooted teeth with relatively straightforward canal morphology. Future studies should evaluate AromaRoot-Laser in more complex anatomical scenarios, including curved and multi-rooted teeth, as well as in vivo conditions. Clinically, the use of AromaRoot-Laser may provide a safer and more effective method for thorough root canal debridement, minimizing the risk of smear layer-related microleakage and postoperative complications. Its promising results in the apical third highlight its potential for improving treatment outcomes in endodontics, particularly when standard irrigants are insufficient.

### 4.4. Statistical Validation and Clinical Implications

The statistical analysis, including two-way ANOVA [F(3, 6) = 101.96, *p* < 0.0001 for treatments; F(2, 6) = 69.12, *p* < 0.001 for sections; F(6, 6) = 11.34, *p* < 0.01 for interaction] and post hoc Tukey’s HSD, confirms these regional differences and treatment effects, as visualized in Figure 2. The non-normal distribution (Kolmogorov–Smirnov, *p* < 0.05) suggests caution in interpreting ANOVA results, potentially favoring non-parametric tests like Kruskal–Wallis, but the findings align with SEM, score distribution (Figure 1), and bar chart observations. The high Kappa agreement (κ > 0.85) and Chi-square tests (*p* < 0.05) validate the reliability and significance of smear score variations across sections. The standard deviations further highlight consistency, with EDTA showing the lowest variability coronally (±2.5), while the apical third exhibited greater variability (e.g., ±11.5 for AromaRoot), reflecting anatomical challenges.

Clinically, these results suggest that AromaRoot with 980 nm laser activation offers a superior alternative to EDTA, especially apically, addressing limitations of traditional irrigants like dentin demineralization. AromaRoot’s QAC-based, biocompatible, and aromatic properties, combined with laser-induced mechanisms (shock waves, vapor bubbles, ROS), minimize cytotoxicity while enhancing cleaning, as seen in Poggio et al. (2011) with photoactivated disinfection [25]. However, the poor performance of AromaRoot used alone (Scores 3–4) indicates its reliance on laser activation, while EDTA-Laser’s reduced apical efficacy (Score 3) suggests laser alone may not sufficiently enhance EDTA’s chelating action in complex regions. Figure 2 visually emphasizes these differences, with AromaRoot-Laser consistently outperforming other treatments, particularly apically (*p* < 0.001).

### 4.5. Study Strengths and Limitations

This study presents a novel irrigation strategy integrating a QAC-based irrigant (AromaRoot) with laser activation, demonstrating strong antimicrobial and smear layer removal performance, particularly in the apical third where conventional irrigants are often limited. A key strength is the dual assessment of microbial efficacy and smear layer removal using standardized protocols and quantitative SEM evaluation. In addition, the study incorporated strict inclusion criteria for tooth morphology, randomized grouping, and validated scoring by blinded examiners, enhancing reproducibility.

However, several limitations must be acknowledged. First, the study was conducted entirely in vitro using extracted single-rooted teeth, limiting its direct generalizability to the clinical setting. The absence of physiological conditions such as blood, immune responses, and canal curvature may affect translational relevance. Furthermore, while AromaRoot’s performance was promising, it was tested against only a single bacterial species (Enterococcus faecalis), and future studies should explore multispecies biofilms. Additionally, while specific pulsed laser parameters were used to activate the irrigants, potentially mitigating thermal damage, this study did not evaluate the physical effects of this laser mode on the dentin surface, such as potential micro-structural alterations, defects, or the uniformity of energy distribution along the canal wall. Comparing different laser parameters and modes (pulsed vs. continuous wave) regarding both efficacy and potential dentin effects could be explored in subsequent work. Clinical trials are warranted to validate these findings in vivo and optimize irrigation protocols for broader clinical adoption.

## 5. Conclusions

This study provides compelling evidence that the SMART method, which integrates AromaRoot with 980 nm diode laser activation, significantly enhances smear layer removal and bacterial reduction in root canal therapy. The bacterial reduction outcomes demonstrate that AromaRoot with laser activation (Groups 3 and 4) achieves near-complete elimination of *Enterococcus faecalis* (98.78% and 99.00% reduction, respectively), surpassing traditional 5.25% NaOCl (98.34%) and NaOCl with laser (98.50%) (*p* < 0.001). This superior antimicrobial efficacy, driven by laser-induced RNS disrupting bacterial membranes, underscores AromaRoot’s potential as a safe, biocompatible, and aromatic alternative, minimizing cytotoxicity while addressing persistent endodontic pathogens.

For smear layer removal, AromaRoot-Laser consistently outperforms other treatments across the coronal, middle, and apical thirds, achieving mean scores of 82.75 ± 5.9, 71.37 ± 1.96, and 78.75 ± 3.4, respectively, corresponding to Scores 1–2 (minimal to moderate smear layer) and 78.5–81.0% open tubules. SEM micrographs, smear score distributions, and statistical bar charts confirm AromaRoot-Laser’s superiority, especially in the anatomically challenging apical third (78.5% open tubules, Score 2), where it surpasses EDTA (64.5%, Score 2), AromaRoot alone (43.0%, Score 4), and EDTA-Laser (34.0%, Score 3) (*p* < 0.001). The laser’s generation of shock waves, vapor bubbles, and ROS enhances AromaRoot’s QAC-based action, solubilizing smear layer components and overcoming anatomical barriers, while EDTA remains effective coronally (89.0%, Score 1) but less so apically.

These findings highlight AromaRoot-Laser’s dual efficacy in bacterial disinfection and smear layer removal, offering a safer alternative to NaOCl and EDTA by reducing dentin erosion and cytotoxicity risks. The consistency of AromaRoot-Laser, evidenced by low standard deviations (e.g., ±1.96 to ±5.9) and high smear score distributions (65–78% Scores 1–2), suggests its reliability for clinical application, particularly in challenging root canal regions. However, the study’s in vitro nature, variability in apical outcomes, and reliance on laser activation for AromaRoot’s efficacy indicate the need for in vivo validation, optimization of laser protocols, and exploration of biofilm resistance to confirm long-term outcomes. These results support SMART as a promising, dentin-sparing alternative to standard irrigants like NaOCl and EDTA, highlighting its clinical relevance by providing enhanced antimicrobial action and effective smear layer removal while minimizing risks such as dentin erosion and cytotoxicity. Further clinical validation and exploration of optimized laser protocols are warranted to establish SMART as a standard practice in endodontic therapy, ensuring its efficacy, safety, and reliability in routine clinical settings.

## Figures and Tables

**Table 1:** Descriptive statistics of the bacterial reduction (%) of the different groups for *E. faecalis*.

Group	Treatment	Mean (%)	SD (%)	Min (%)	Mdn (%)	Max (%)	Post Hoc
1	5.25% NaOCl for 3 min	98.34	1.33	96.03	98.34	99.5	A
2	5.25% NaOCl + 30 s and 60 s laser	98.5	1.54	97.23	98.5	99.45	A, B
3	AromaRoot + 60 s laser	98.78	1.4	97.08	98.78	99.65	C
4	AromaRoot + NaOCl + 60 s laser	99	1.2	97.75	99	99.85	C

Groups with the same letters are not significantly different (*p* < 0.05, Mann–Whitney post hoc test).

**Table 2:** Percentage of smear layer removal or open dentin tubules by different irrigation techniques across coronal, middle, and apical sections.

Groups	Treatment	Coronal (%)	Middle (%)	Apical (%)
1	Control group (17% EDTA)	89	69	64.5
2	AromaRoot	51.5	55.2	43
3	EDTA with laser	78.5	58.5	34
4	AromaRoot with laser	81	71.3	78.5

**Table 3:** Means and standard deviation (SD) score values of the smear layer for different activation techniques in the apical, middle, and coronal thirds.

Groups	Coronal (Mean ± SD)	Coronal (Min)	Coronal (Max)	Middle (Mean ± SD)	Middle (Min)	Middle (Max)	Apical (Mean ± SD)	Apical (Min)	Apical (Max)
1	88.75 ± 2.5	84	93	68.81 ± 6.5	58	81	64.50 ± 4.8	57	72
2	51.37 ± 3.2	46	57	55.50 ± 4.8	48	63	44.75 ± 11.5	28	63
3	78.87 ± 3.7	73	85	58.50 ± 2.6	54	63	34.50 ± 4.8	27	42
4	82.75 ± 5.3	74	92	71.37 ± 1.96	68	75	78.75 ± 3.4	73	85

## Data Availability

All data are available within the article.

## Abbreviations

The following abbreviations are used in this manuscript:

EDTA	Ethylenediaminetetraacetic acid
NaOCl	Sodium hypochlorite
LAI	Laser-activated irrigation
QACs	quaternary ammonium compounds
RNS	Reactive Nitrogen Species
ROS	Reactive Oxygen Species
SMART	Synergistic Microbicidal and Activated Root-cleansing Technique
